# Supplementary material for: What are the barriers to, and enablers of, working with people with lived experience of mental illness amongst community and voluntary sector organisations? A qualitative study
Source: PLoS One. 2020 Jul 2;15(7):e0235334. doi: 10.1371/journal.pone.0235334 (PMC7332084; doi:10.1371/journal.pone.0235334)
Supplement: S1 Material — (DOCX) [file pone.0235334.s001.docx]

Supplementary Material

# Methods

Initial recruitment took place within the MARCH mental health research network. The MARCH network is one of eight UKRI funded mental health research networks, and has over 1,000 members, including community organisations, policymakers and researchers. MARCH focuses on social, cultural and community assets – which includes the arts, culture, heritage sites, libraries, green spaces, community centres, social clubs, community associations and volunteer groups – and the role they play in enhancing public mental health and wellbeing, preventing mental illness, and supporting those living with mental health conditions.

Focus groups both in and outside London were planned to ensure that experiences of regional, or rural, groups were included. Interested potential participants received an information sheet detailing the research background, purpose and aims ahead of data collection. Consent forms were also sent ahead and any questions or discussion via email or telephone call invited.

The topic guide was developed by LB (Research Associate, trained in qualitative methods) and DF (Associate Professor and Principal Investigator) and focus groups were led by LB and two colleagues from the Department of Behavioural Science and Health at UCL. All participants were asked to not repeat others’ responses outside the group. A further ten representatives who had previously agreed to participate withdrew ahead of the groups, for reasons including pressure of work, under-staffing, distance to travel, and illness. No participants withdrew their data from analysis after the focus group took place. Focus groups in London were held at University College, London; the South-West and South Coast groups were hosted by community organisations.

One researcher (LB) undertook the familiarisation and initial coding stages. Each transcribed focus group was read fully at least once to be as familiar as possible with the data before formally coding. Having re-read the transcripts, an initial list of codes was produced. Codes were revised in the light of new data where necessary, and an ‘audit’ trail of code development between each focus group transcript was kept (Ranney 2015). The codes and process used to reach them, and development and review of interpretive themes, was then discussed and verified with other researchers in the team.

# Topic Guide

Opening question

1. Could we start by asking about what type of community activity your organisation specialises in and whether you’re currently doing any work with people with lived experience of mental illness?

Three main questions

1. For those of you already working with people with lived experience of mental illness, what was it that first motivated you to run projects for people with lived experience?
   1. What is it that still motivates you to take part?
2. What factors, skills or characteristics that you think your organisation has in order to be able work in this area? What makes it able to engage/ successful in engaging?
   1. Or if you’re not leading this work, are there things you feel you’re missing/ Are the qualities that you think are needed?
3. Are there factors in the wider environment (outside the immediate organisation) that help with, or hinder, this work? What opportunities might you be waiting for before engaging more?

Closing questions

1. Now you’ve started working with people with lived experience of mental illness, do you think you will ever stop? Why/why not? OR If you’re not already engaged, what do you think might change that and make you want to engage?

# Coding Manual

## Motivation

### Mental Health Motivation

#### Responding to needs

Motivated to go into this work by the mental health needs of others. Wanting to help, and responding to perceived gaps in services. Often underpinned by own lived experience of mental illness.

Codes: Helping others, Responding to a need, Filling a gap in provision, Gap in provision for those with physical and mental illness, Lived experience as motivation

- - “We work closely with another organisation called [org name]. Some people come to us that don't want antidepressants or to go to the hospitals, they just want to do something. And we refer them, they are working with [org name], they’re doing massage, acupuncture, singing, doing body cycle therapies, and they are getting better.”
  - “And those things, certainly what drives me and people we don't actively… It's interesting what you're saying actually, we don't actively seek people with mild to moderate mental health issues, but that is mostly who approaches us.”
  - “So what was the motivation? What kind of made you sort of want to get involved in this area?” SP: “It’s a mixture just sort of knowing that being creative amidst all the people is, you know is good for your health and wellbeing and knowing there’s very little service provision out there especially in the rural area.”
  - “We’re a community interest company. I started that exactly for the reason of supporting people with mental well-being. About eight years ago, I had a breakdown, and I’ve used arts therapeutically to regain some wellness and take that forward.”

#### Witnessing benefits

Continued motivation by seeing the benefits to participants from taking part in the group/ organisation or activity.

Codes: Benefits for users, Seeing the benefits, Research evidence

- - “And you know, and the participants just saying what I felt at the beginning they would say, but it’s different when you actually hear it or you actually add up the work and you go, oh my God, that’s incredible.”
  - “Just is witnessing the change in peoples' lives and it's not every year to see and to be unable to come to class to [unclear] either performing or sharing their achievements. It's wonderful”
  - “The evidence being reported back to us that mental health, wellbeing, confidence, feeling part of a community was being reported to us over and over. And it seemed as though there was something kind of magical about making music and singing for these young people that was insulating them against longer term mental health issues. And we were getting these observations from the teachers, school leaders, parents, from the young people themselves. Tons of anecdotal evidence and so much of it that it ceases to be anecdotal, of young people saying when I sing I feel so much better. I feel connected with my friends. It makes me feel happy, there’s no pressure on me.”

### Community Motivation

#### Expanding inclusion

Opening up community and cultural activities to a wider and more diverse group; includes the opportunity to address stigma.

Codes: Addressing stigma, Speaking against injustice, Support for artists as motivation, , Intrinsic value of creativity, Personal taste in type of work

Sample data:

- “ But because I was working in music at the time and because all of [org name]’s work is focused on what happens outside of school, what happens to the kids who miss out? What happens to the kids who lack opportunities? What happens to the kids who don't engage with school and who are perhaps being educated outside of mainstream schools?”
- “And I’m always looking at who is an unusual audience, where could we make a show? Why are we doing it? It has to have some kind of social or political agenda for me to feel interested in it.”
- “We set up a community mental health centre which had a café. So it was really about integrating people back into community. Addressing stigma, breaking down… Giving people a sense of purpose and self-worth”
- “So that's their whole thing so I think stigma is a really big… Breaking down stigma is a big thing.”
- “I think we used to think more big picture but then if you start thinking, what if people weren’t creative and what would we lose, what would be damaging to the way we live and function as a society?”
- “SP2 Coming back to the motivation thing, I think what keeps us motivated as an organisation is that I really believe that we are offering something that isn’t being offered elsewhere. So we have a music service that is obviously much bigger than we are and delivering music education, but I think that we’re delivering something that’s a bit different, and more than music.

MO That’s because of your focus on inclusion?

SP2 I think so and our experience in understanding and working with the person or the people in front of us rather than working to a plan on a piece of paper. We’ve got to deliver to this person, whoever they are. It is a completely different approach.”

#### Increasing asset sustainability

Includes economic motivation. Becoming involved in this area allows the asset/ group to access additional areas of funding and work opportunities that might allow improved sustainability and growth.

Codes: Economic motivation, Lack of funding creating a gap, Increasing asset sustainability, ‘Everybody’s doing the mental health thing now’

Sample data:

- “So it’s the merging of technical expertise and experience, and that’s been running since 2013 and developed as it’s gone along. And it’s become more of an organisational priority for us. I think again for all the reasons you mentioned because it’s something we’re all more aware of now, obviously it’s something that there is funding available for”.
- “So actually there was an economic imperative to engaging with the free drama workshops for people. .. because he could get public health money to do it”
- “I want to be clear that the drivers for the things that we do to sort of deliver on the kind of vision that I was talking about are things like funding of course, funding streams”

## Capability

### Developing skills

#### Understanding statutory responsibilities

Running the asset, CIC or charity often requires a range of potentially new skills and knowledge, such as requirements for running businesses, safeguarding, GDPR and grant applications

Codes: Worries about safeguarding and regulations, Running a business, business skills, skill set needed

Sample data:

- “I think some recent changes to the law over the last few years have exaggerated that because under health and safety laws now you can be found individually responsible in an organisation and sent to jail and that didn't used to be the case. And so the company secretary could go to jail for up to five years which are quite significant amount of time for an offence, for want of a better term, nobody wanted to happen and tried their best to ensure it didn't happen. But by opening your doors to people who otherwise need of that help, there's a challenge there.”
- “And so within the school if something goes wrong there's somebody there other than us who needs to deal with it. So that makes it much easier actually. If we were to start doing stuff totally independently I think that is a seriously different ball game.”
- “ I don't like if that were a part of a company’s house as a limited. I just… That’s crazy, isn’t it, amidst all that rigmarole, but…

PA3 You have to do your annual statement every year, and…”

- “I think a lot of organisations either can’t grasp that. They certainly can’t write a policy for it, but also actually it’s a real challenge to work with on a day-to-day basis because sometimes that means you cannot plan… “
- “ I’m still recovering from GDPR.”

#### Support and training for staff

The ability to train and support staff in the way they need, including front-line and freelance staff.

Codes: Ability to provide supervision for staff, Training of staff and practitioners, MHFA training, Lack of support for freelance practitioners

Sample data:

- “We may then go to work in the field and they work in projects they have supervision as well so they're not on their own with the community groups that they are working with.”
- “I think it’s really dangerous, I really do. I think there’s immense risk that people who are not really responding to this appropriately. And we did an intervention evaluation in 2016 that looked at how we do things and why it might work. And the focus that we had was support, and the ability of us to have a very strong core that then allows flexibility was absolutely key to the success of the organisation.”
- “SP6 We give our musicians sort of arts and health and arts and wellbeing training as part and so we bring in the latest evidence thinking and then as I say we feed that back into the way we develop their practice. So they understand the context of the practice. We definitely do it better than we are doing but that’s certainly what we aim to do.”

SP4 Yes, it would be wonderful if that was the norm. I think it’s great to just try it, yes.”

- “But also having the actual medical, being able to refer them, you know if there is crisis, being able to know what to do with that because you’re not necessarily equipped to be able to deal with it. So I think there’s a lot of, yes support needed about that, support for the staff.”

### Understanding boundaries

#### Coping in the moment

The ability to cope with potentially vulnerable people’s needs, and to manage response to this, including the desire to maintain an identity as an artist/ practitioner, rather than mental health worker.

Codes: Understanding boundaries, Challenging nature of participants, Conflicting needs of participants, Therapeutic not therapists, Safe environment

- I think generally there’s probably not been that many bad experiences so people feel they can cope. But if this starts to become much more wide-scale issues will start to surface I just wonder how that skills gap is going to be plugged if it is just funnelling from one to the other without enough planning and preparation.
- “And there just seems to be a lot of this enthusiasm now that like social prescribing is somehow going to save the arts sector and it’s not really at all and part of that is it’s sending people with complex needs to people who aren’t properly trained. I think there’d be very few organisations that feel that they’re properly trained up and capable of dealing with a range of mental health issues. It’s such a vast and difficult field that as any sort of arts practitioner or organisation to be properly equipped for that is a real challenge.”
- “At the outside edge of this we're very clear that we are not doing anything other than providing an activity. So there's no pretence of therapy although the outcomes may be therapeutic the output or the activity itself is not. And so we are qualified to do what we do because my facilitators have MAs in applied theatre or I'm a professional theatre director. And so there's that is just a fact.”
- “We’re not therapists, but we are working therapeutically.”
- “And I think one of the dangers is there is this whole spectrum of work isn’t there from people who are specifically music therapists working with people with mental illness and they have very specific boundaries and supervision and then you’ve people who are say running choirs that happen to be community choirs that happen to have people with mental health issues in them and they’re actually doing this for them but don't realise they’re doing it and they’re everything in between you know? Where do we or where do arts organisations, you know where do we look for the practitioners, you know where are the boundaries?”

#### Burn-out

The feelings of burn-out and feeling overwhelmed, in response to blurred boundaries, skills shortage, or having to fulfil a number of roles.

Codes: Burn-out, Feeling overwhelmed

Sample data:

- “So one of our taglines is we will never work alone. So again it’s one of these bizarre things that we wanted to do something but then we were actually burning ourselves out, and there was no point in doing that. So it’s slowly how for me as a culture, because we will be here for a few years and then we will move on and other people will take over this amazing organisation. So for me it’s very important as we start these baby steps to ensure that we actually are looking at our own health and our own wellbeing. And we have a lot of people around us.”
- “MO1 Do you feel that you have the capability to work with the community? Do you feel supported?

PA2 No. We are actually just about to pilot our first social prescribing funded activity, something that we run on a day-to-day basis which is called [Project name] where we offer the space for people to come in. And that is a supported activity and we are open five days a week. And people are welcome to come in all the time. I don't feel like I have the capacity to do that every day, sometimes it can be really hard.”

- “I’ve gone through a depression, and I also have memory issues, close to suffering a bit from post-traumatic stress disorder from running this place, running this venue and all the hassle and all the shit we’ve had to put up with, with people not recognising that everything that we are doing around the table right now is valid and has a weight to it and actually helps the service providers who have so many waiting lists that these people can’t access.”
- “And that’s why people are under qualified in terms of capacity. That’s why people burn out. All of these things are happening, so we need to have a very, very grown up conversation about the fact that it’s actually okay to say no. Like even when we burn out at work and we say, oh you can do that, I say no we can’t do that”
- “But again this is when you have to go actually we can’t sustain it, we do not have the capacity, not just the staffing capacity or the financial but again the emotional capacity to keep that going. And we are, you know as our momentum is building this is the lesson we very much learn is going where do we put our efforts into? What’s the most important thing at the moment? Because we cannot do everything thoroughly and well”

### Offering something different

#### Responsive

The ability to offer something person-centred, empathetic and responsive to people’s needs.

Codes: Understanding people’s needs, Responsive, Open Communication, Person-centred, inclusive, Diversity of assets and staff, ability to reach those excluded from services, Bringing people together, Local community based knowledge

Sample data:

- ”And diversity and quality and kindness and tolerance are much wider values that become an alternative narrative. And I think that the concept of inclusion and the work that we do with inclusion really fuels that opportunity to talk about that alternative narrative for compassionate leadership”
- “I found that the qualities … are really about communication skills and empathy and really being passionate about your art form because then you're really inspiring if nothing else, what we're doing.”
- “So it’s making as comfortable a space as possible and then using the skills and experiences that we’ve built up and having artists that that we’ve always got people’s, like really good people skills as well as the art skills.”
- “And when I’m recruiting music practitioners I am looking for something quite special. I am looking... the most important thing is that they’re able to connect with people.”
- “Something we’re passionate about, we motivate you to wake up and want to go out there. And you are not just decorating cakes actually but we are talking to see whether there is a cake college where we can enrol her, where she can do that. So that is the way we do our social prescribing within our service.”

#### Innovative

The innovative offer of the asset for people with lived experience of mental illness, including offering creative opportunities.

Codes: Innovation, a different way of doing things, giving creative opportunity, giving back creative control

Sample data:

- “I think one really important thing is not being afraid of change actually, and being allowed to fail. Those are really important. And especially I think when things aren’t always going well, to be allowed to fail and learn from that in a safe way.”
- “ It strikes me sometimes that too much background almost promulgates the status quo of doing a certain thing in certain ways. But we may find with some mental health organisations that they won't trust us because we don't have… It's a bit chicken and egg, that we don't have the experience to show that we get good results.”
- “So it’s really interesting that again, so [name] think of all the people who’ve participated in programmes as their audience. So the core staff go out to at least two to ten sessions and they assume that actually the people participating in the session are their audience and they have a responsibility to have a relationship with them. And I suspect that’s really unusual for the arts organisation doing that now. Of course they are literally their audience. They don't have another audience. They aren’t a theatre with an audience in the building doing some outreach work on the side.”
- “So I think the ability to have those conversations and be dynamic and be able to adapt to things that are happening around you I think is a real strength. I think having, you know being able to be asked by say [name] can you come and deliver a few sessions and for that to happen at least allows people to test the water and if they like it they can move forwards with it.”
- “So although it might seem a bit cosseting to pick them up it doesn’t mean they turn up. Whereas they might not if they feel a bit alone that day. Whereas if a taxi knocks on the door they’ll get in it but they might not somehow. They can’t walk to the bus stop they’re not going to get on the bus with all those people or whatever. Some people do make their own way.”
- “We’re one of 50 in England with us being the only social enterprise model.”
- “So I think having a real range of different styles and deliveries are real assets. Certainly there are so many different people that come to the sessions. I mean just on a day-to-day basis in our organisation different styles appeal to different people. So there’s something about the diversity of practice.”

## Opportunity

### Partnerships

#### Cross-sector collaborations

The opportunity to collaborate across sectors, such as health and education, to provide the service and/ or fulfil the aims of the organisation.

Codes: Collaborations with partners, partnerships, strategic partnerships, staff turnover in partnerships

Sample data:

- “I mean that’s the ideal isn’t it to have a health partner that knows the value of this work that is prepared to work in partnership with you to develop something that meets everybody’s needs and is safe and is evidence-based etc.”
- “Yes I think it’s definitely collaboration and partnerships.”
- “And where it works best is where there’s a really good partnership working.”
- “So you’re looking for somebody that’s a partner or organisation and then you get a good working relationship with that person who understands what you do and understands your organisation and your work and then they move on.”

#### Peer support and learning

Opportunities to learn from, support, and to train, other organisations within the sector.

Codes: Learning from other organisations, Training others, dissemination, Mutual trust, Ability to make connections

- “I think it’s really important that we train other people though. I really want to not be the person that everything depends on. I really want that, so the idea with [name] would be that we do it next year in three or four locations in England. And in each of those locations we train the other local artists to then become groups. Because I think that that dissemination is really important. And when you’re talking about protecting what’s yours versus sharing, I think maybe... I really understand the pull and the tension there, but I think maybe an approach is to take ownership of how you share in that you say, I will share it on my terms. !
- “So you’re in charge of how you train other people to do your work, to replicate that rather than this idea of just write it down and I’ll do it. No, I definitely won’t do that but I will do this for you if you really want to know about it.
  - SP2 It’s tricky then if you’re working with a national organisation who are going to then take what I input and they’re going to own the training.
  - SP1 Do you have any say over that?
  - SP3 Can you say no to that?
  - SP2 It’s a bit tricky because if I say no, then I’m...
  - SP1 Can you say not that but this?
  - SP2 Maybe. I am trying to be very open and have honest dialogue with them about it, but I do think it’s an issue. I think that we do want to share and I do think that we need to be training. “
- “So we are aware there are going to be some potential issue with that. We don't want to have to handhold all the way through this because we can’t do that, you know there’s other projects we need to be focusing on. But absolutely that is definitely something we are discussing at the moment and working on how we’re going to be able to, you know and some places will be able to do it better than others, you know others will be struggling. “

#### Appreciation

Being undervalued/ exploited by others. Including being misunderstood/ undervalued by the health sector, or within relationships with larger organisations such as established charities or established cultural assets. Includes believing that you ‘tick a box’ for other organisations.

Codes: Undervalued, exploited, Being a ‘tick box’, Perceived as the ‘other’, Giving away time for free, Austerity, arts as stopgap,

Sample data:

- “ The thing that has been touched on around almost tokenism. We have a lot of creativity and people come into us all the time, we need your people to do what we’re doing. And that’s very challenging because actually it’s about building relationships and trust.”
- “But I don't know. I feel sometimes it’s just because it doesn’t come out of a bottle, it’s not good enough and that’s wrong.”
- “There's a couple of things people have offered us work and I had to say I can't do this because I can't get there unless you're willing to cover my cost. And then, oh well we're only a charity and we could just offer you the work but we can't afford to pay you. And okay and I do lots of that kind of work where I'm giving of my time. It's okay I appreciate that because a lot of people who can't afford to pay but then it's not helping us move on and develop us so that we can do more work.”
- “Where it doesn’t work so well is where we go in, and they barely know that we’re there and we do it in isolation.
  - SP3 You’re a tick box, aren’t you?
  - SP2 Yes.
  - SP3 I don't mean in a rude way, but you know what I mean?
  - SP2 Absolutely, yes.”

#### Cross-sector disconnection

Disconnection between the health sectors and community and cultural assets. Including use of/ lack of a shared language across the sectors.

Codes: Disconnection between the arts and health, Lack of understanding in the medical profession, Speaking the same language, Clash of cultures

- 1. “And then particularly like working for the NHS, how do you fit within their corporate weirdness, because they speak a different language. “
  2. “If you haven’t got either side completely grasping why themselves as well as the crossover works, it’s no wonder that it’s taking a lot of unpacking actually, and I think that’s probably what it does need. It needs people on both sides of the practices to come together in an honest dialogue about what does work, what doesn’t work”
  3. “And for me at one end, I think it’s trying to create more partnerships with local GP people to give them a taste. To say, right come on guys. I know you all work but I’ll do 15 minute writing with you so that you can feel it. Because I think a lot of it is that people don't have experience of it and don't understand it”
  4. “And there’s a huge disconnect between the health care side of things and the artistry side of things. I mean when we were doing that pilot with the GP surgery for the chronic pain, afterwards they were like yes our patients really loved it. We were thinking like a couple of our GPs are really into musical theatre, we were wondering if they could run it.”
  5. “We've just finished the pilot with older people but the thing that I found really difficult is identifying groups that are willing to listen to… And to try what we're proposing. And it's like pulling teeth. And I want to understand how to get around that and I think we've been thinking a lot about why it was like pulling teeth and I think a lot of it had to do with the fact that it's very different”

### Implementation

#### Commissioning

Commissioning politics and processes. Opportunities for commissioning are perceived as being reliant on ‘who you know’ and regional differences in approach to this area.

Codes: Lack of clear commissioning pathways, Accidental commissioning, Funding for friends, Funding on reputation, How to overcome the ad-hoc approach

- “SP1 And also how the NHS and the arts organisations get to know each other. Because when we were commissioned to do the wellbeing groups that happened completely by chance, completely.

SP2 It often does.”

- “ And then I got a call from her and she said, would you come to a meeting with me and this other person? And it turned out that this person had had the budget to commission us to run these groups. But I would never have known how to get there if it wasn’t for just someone who was a friend.”
- “I think a big thing is what you were saying, it’s about who you know. It really is. Yes. Just those personal connections really go a long way and can get you in the door. So that’s making use of that really but it’s not fair.”
- “If you get onto the golden list, you’re all right.”

#### Sustainability

The sustainability of, and threat to, the assets through the lack of/ difficulty in obtaining and maintaining funding.

Codes: Sustainability and threat, Postcode lottery for funding, Funding support for staff, Funding and resources, Being able to fund what participants need, ‘Need is rising and funding is falling’, Not wanting to fund arts and health, Keeping it free and accessible, Cost of projects, Funding link workers instead of groups

- You have a responsibility then to be able to create the systems in place to make sure that that’s okay. And usually that’s not invested in. Those conversations are not invested in. The music sessions are invested in, you get money from funders but the funders are not seeing the wrap around care that happens in inclusive settings and if you’re dealing with mental health. And I think that’s a really interesting issue to draw out in terms of asset based approaches.
- “But also for the NHS this is meant to be a cost effective for them to be referring people to our organisations. But yes, there’s not the acceptance or recognition that this is costing our organisation money. I think it’s slowly, from the reading that I’ve done, I think there is a slow recognition that money needs to come down to the actual organisations.”
- “It's the same with me I think because I can only do this part-time at the moment. I'm lucky enough to have a part-time day job that pays the bills otherwise I don't know where I'd be. However, I can't afford to pay any staff at the moment to help me develop the business because I'm not earning enough and like I say I need people who will come on board and help me develop it, give me the funding in order for me to go that next step so that I can do more of this work.”
- “If there are funders offering funding specifically this type of work that should be built in within the funding application process how you are going to support the practitioner. That mechanism must be in there. But there’s going to have to be an education for funders as well because you know when projects are being written and funding applications are submitted, you know the costs get pushed down. And unless funders understand this is being an intrinsic element of the ethically, morally, legally safe practice or correct or best practice one might say, then it needs to be funded”

#### Transparency

Understanding and navigating working in this field, particularly with reference to social prescribing and link workers in the health sector. This includes the difficulty of encouraging and receiving referral from GPs, via informal prescribing or social prescribing

Codes: Not understanding social prescribing, Worries around social prescribing implementation, Navigating social prescribing, Quality control of social prescribing, reluctance to prescribe/ refer, social prescribing as a distraction, receiving referrals, Informal prescribing by GPs

- “One of my ladies was really buoyed up and she decided to join another choir. And within two weeks they told her she sang flat and that she wasn't allowed to come back and she was destroyed. And so my concern was with social prescribing is yes I run a choir, signpost people to me. And actually we're not… The people aren't empathetic they're not the right people, we've got no quality control of that.”
- “You know? Who is responsible in that and it’s all got to be ironed out, I guess, but those are some of the things that I worry about.”
- “Are the NHS going to facilitate this process or is it that we always have to be going to them and trying to fit in with what they’re doing? I think that’s quite difficult, you know?

SP2 I think there are examples around the country of both of those happening.

SP3 Yes. We need it to be consistent.

SP2 There isn’t consistency, yes.”

- “But they really need to be trained or they need to be very well supported in terms of understanding as you were saying, the landscape into which they are signposting. And it is signposting, it’s not going to be referral. The scale at which they’re working they cannot do referrals. They’re just going to be saying go there.”
